# Comparing MRI volume measurement techniques for pituitary macroadenoma: Investigating volume reduction and its relationship with biochemical control

**DOI:** 10.25122/jml-2022-0196

**Published:** 2023-07

**Authors:** Ibrahim Hani Hussein, Abbas Ali Mansour, Nabeel Abdulateef Jameel

**Affiliations:** 1 Department of Diabetes and Endocrinology, Faiha Specialized Diabetes, Endocrine, and Metabolism Center (FDEMC), Basrah, Iraq; 2 College of Medicine, University of Basrah, Basrah, Iraq

**Keywords:** pituitary adenomas, functioning pituitary adenomas, NFPA, pituitary tumor volume

## Abstract

Pituitary adenomas are one of the most common types of primary intracranial tumors. Measuring pituitary adenoma volume is fundamental for effective management. This study aimed to assess the reliability of the ellipsoid method in comparison with the perimeter method for measuring pituitary macroadenoma volume. In addition, we investigated the correlation between adenoma size reduction and biochemical control in functioning adenomas. This was a retrospective cross-sectional cohort study including 113 patients with pituitary macroadenomas. MRI was obtained for volume measurement by ellipsoid and perimeter methods using two types of DICOM viewer software. Both ellipsoid and perimeter methods exhibit positive, strong, and significant correlations in pituitary macroadenomas in pre-treatment and post-treatment volume (Spearman correlation coefficient 0.95, p-value <0.0001). There was no significant difference in the mean post-treatment pituitary adenoma volume measurements utilizing the ellipsoid and the perimeter methods in different treatment modalities. There were significant differences in the pre-treatment volume measurements between the two methods, both in NFPA and prolactinoma. No correlation was found between volume variability measured by ellipsoid and perimeter methods and the degree of hormonal control in functioning pituitary adenomas. Both the ellipsoid and perimetric methods can be utilized for pituitary adenoma volume measurements as they demonstrate a strong and positive correlation. However, it is important to note that the ellipsoid method tends to result in overestimated tumor volume. There was no correlation between the adenoma size reduction and the degree of biochemical response in functioning adenomas.

## INTRODUCTION

A pituitary adenoma is one of the most common types of primary brain tumor, making up 14% of cases, and its prevalence in the general population is as high as 17% [1, 2]. Functioning pituitary adenomas encompass various classifications based on the hormonal activity exhibited by the tumor. These classifications include lactotroph adenoma, somatotroph adenoma, corticotroph adenoma, and thyrotropin adenoma. On the other hand, adenomas that do not produce any hormones are categorized as clinically non-functioning adenomas (NFPA). Pituitary adenomas can be classified based on their invasiveness and the presence or absence of suprasellar extension, which can be determined through meticulous neuro-radiological examination. The widely adopted classification proposed by Hardy in 1972 [3] further categorizes them as noninvasive or invasive, with or without suprasellar extension. While these classifications have been used for many years, it is worth noting that they rely on staining methods that may not strongly correlate with the hormonal activity of the tumor. However, this classification remained valid until the last decade when the World Health Organization (WHO) introduced a new classification for endocrine tumors of the pituitary gland in 2017, known as the fourth edition [4].

Over the past few decades, the field of pituitary imaging has witnessed significant advancements. Magnetic resonance imaging (MRI) has become the modality of choice for assessing the hypothalamic-pituitary axis based on its ability to delineate and discriminate both the pituitary gland, its pathologies, and the surrounding structures with a greater benefit over the conventional computerized tomography (CT scan) in patients necessitating follow-up without risk of radiation [5, 6]. Furthermore, MRI provides robust information regarding the degree of cavernous sinus invasion and the extent of optic chiasma compression upon which endocrinologists base their clinical and surgical decisions. For adequate details and delineation of the pituitary pathology from the normal pituitary gland, several important MR sequences have recently been added.

Both Hardy and Knosp described qualitative methods for assessing adenomas, but these methods have different clinical importance in assessing the degree of cavernous sinus invasion, suprasellar extension, and chiasmal compression. Furthermore, these methods are insufficient to assess the extent of the disease and the response of volume to various modalities of therapy [7]. Several methods have been described to measure pituitary adenoma, including the oldest linear (maximal dimension), the most modern three-dimensional (3D) volumetric MRI software, and the semi-automated method, which carries variable inaccuracies due to the tumor growth behavior even though it is simple and less time-consuming [8, 9]. Extension of pituitary adenoma and the response to treatment with size reduction is unpredictable. A study of the maximal dimension of adenoma alone is insufficient. A comprehensive method for measuring adenoma volume is necessary to assess the extent of tumor involvement and response to therapy [10, 11].

The primary objective of this study was to assess the reliability of the ellipsoid method in comparison with the perimeter method for measuring volume in cases of pituitary macroadenomas. The secondary objective was to determine the correlation between biochemical control of functioning pituitary adenomas and volume reduction.

## MATERIAL AND METHODS

### Study design and participants

**Figure 1:**
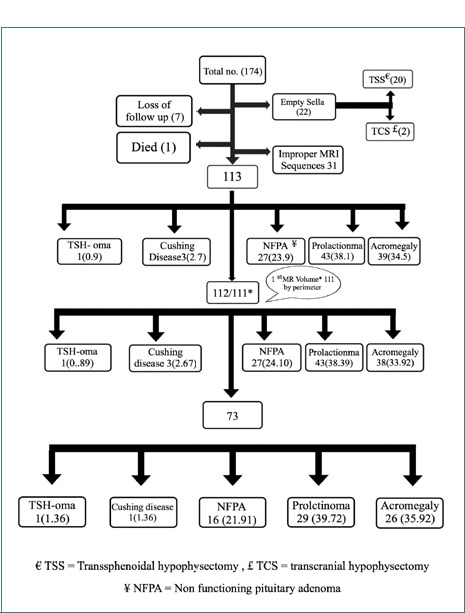
Schematic representation of the studied patients

This cross-sectional retrospective cohort study was conducted at Faiha Specialized Diabetes, Endocrine, and Metabolism Center (FDEMC,) a tertiary center in Basrah, over one year, from October 2018 to October 2019. We enrolled patients with pituitary macroadenomas, including somatotroph adenoma (acromegaly), lactotroph adenoma (prolactinoma), and clinically non-functioning adenomas (NFPA), who presented at the center during the designated study period. Figure 1 provides a schematic representation of the study details. We excluded patients with a history of pituitary surgery and missing preoperative MRI films, patients with adenomas less than 10 mm in diameter, pregnant patients, hypophysitis, and craniopharyngiomas.

### Clinical evaluation

Examination, including height, weight, and body mass index, were performed for all patients, stigmata of acromegaly, florid features of Cushing syndrome, and hypogonadism. Hypopituitarism was defined as the compromise of more than two hypothalamic-pituitary axes. All definitions of pituitary adenomas and their complications were previously established in a study from the same center [12]. In cases of pituitary adenomas that secrete more than one hormone, typically prolactin (PRL) or growth hormone (GH), classification was based on the predominant hormone secretion, taking into account associated signs and symptoms.

### Radiological assessment

Pituitary-directed dynamic MRI with gadolinium enhancement was performed using the appropriate protocol for all patients regardless of whether they were previously diagnosed with pituitary adenoma or were newly identified cases [13]. Subsequently, a second MRI was performed at suitable intervals of at least three months to detect any changes in tumor volumes, and the period of follow-up was considered as the period between the first and second MRI scans. Imaging scans were obtained using a 1.5 Tesla MRI machine. We used a RadiAnt DICOM viewer (64-bit) as a DICOM viewer of choice in the measurement for ellipsoid measurement and an Onis DICOM viewer for the measurement of the perimeter method.

### Volume measurement

Tumor volume was measured in cubic centimeters using 2 methods, the ellipsoid and the perimeter or planimetry (the gold standard method in this study).

The ellipsoid method was calculated mathematically using the formula 4/3 π (a.b.c), where a represents the depth taken in the midsagittal T1-weighted midpoint in the maximal dimension, b represents the width in the transverse coronal T1-weighted image, and c represents the height in the vertical coronal T1-weighted section [14]. The perimeter method involved manually outlining or tracing the adenoma slice by slice, dividing it into multiple known slices, multiplying each slice's surface area by the slice thickness in the T1-weighted image, and summing the surface areas of the traced slices [15].

### Volume variability and adenoma growth

Volume variability was calculated as a percentage using the formula: |V1–V2|÷V1×100, where V1 represents the first volume, and V2 represents the second volume.

Adenoma growth was determined by detecting any increment of a few millimeters (i.e., ≥1 mm) in the size of adenomas in any dimension detected on the second MRI scan using the methods mentioned and vice versa.

### Biochemical evaluation

Morning blood samples (10 ml of venous blood) were collected from all patients after an 8-12 hours fast. The serum was then analyzed for hormone levels, including prolactin (PRL), growth hormone (GH), and insulin-like growth factor (IGF) basal measurements. GH and prolactin were measured using electrochemiluminescence (ECL) Cobas e 411 Roche^®^, Elecsys. Acromegaly control was defined as random GH levels ≤2.5 ng/mL in early morning samples [16], while prolactinoma control was defined as achieving serum prolactin levels of ≤30 ng/mL in early morning samples for both sexes [17].

### Statistical analysis

Statistical analysis was performed using the Statistical Package for Social Science software (SPSS, Inc. Chicago, IL, USA) version 25. Continuous variables were presented as mean ± standard deviation (SD), while categorical variables were summarized as numbers and percentages. The normality and distribution of the values were assessed using Kolmogorov–Smirnov test. The association between categorical and continuous variables was done using an independent student’s t-test, whereas Chi-square (x^2^) and Fisher’s exact test were used for the association between two categorical variables for multiple demographic features among different adenomas. We used a paired t-test to evaluate the significant difference in the mean volume between the ellipsoid and perimeter methods in the pre-treatment and post-volumes among different pituitary macroadenomas. Correlation between two methods of volume measurement in different adenoma types using different modes of treatment were carried out by Spearman rank correlation test, and the comparative test was used for adenoma size variability and biochemical values for degree of control. A p-value of <0.05 was considered significant.

## RESULTS

### Demographic characteristics and adenoma distribution

113 patients with pituitary adenomas were enrolled in the study, with a mean age of 42.16±13.58 years (Table 1). Prolactinoma was the most common type of adenoma, affecting 43/113 (38.1%) patients, followed by acromegaly in 39/113 (34.5%) patients, NFPA in 27/113 (23.9%) patients, Cushing disease in 2.7%, and thyrotropin-secreting pituitary tumors (TSH-omas) in 0.9% of patients. The gender distribution was relatively equal, with men constituting about 50.4% of the studied patients, with a 1:1 men-to-women ratio. No significant differences were found in gender, age, systolic blood pressure, or diastolic blood pressure differences between the groups.

**Table 1:** General characteristics of the patients with pituitary adenomas

Descriptive character		Total No (%) 113(100)	Acromegaly No (%) 39 (34.5%)	Prolactinoma No (%) 43 (38.1%)	NFPA No (%) 27 (23.9%)	Cushing disease No (%) 3 (2.7%)	TSH -oma No (%) 1 (0.9%)	p value
Men No (%)		57 (50.4)	23 (59)	20 (46.5)	12 (44.4)	1 (33.3)	1 (100)	0.53
Age (years) mean ±SD		42.16±13.58	43.28±13.3	39.05±14.04	45.56±14.64	39.00±4.58	50.00	0.34
SBP (mmHg) mean ±SD ^ș^		133±22.03	133.24±25.37	134.31±21.75	130.12±16.23	147.67±30.2	122.00	0.71
DBP (mmHg) Mean ±SD		83±13.58	85.95±15.98	80.40±12.00	83.28±11.85	94.00±5.29	70.00	0.18
BMI ^έ^ Mean ±SD		31.82±6.37	30±4.69	35.15±7.22	29.24±5.19	31.70±3.00	27.300	0.0001
DM ^£^ No (%) total (102)		21 (20.58)	10/36 (27.77)	7/39 (17.94)	4/23 (17.39)	0		0.26
Duration of follow-up (months) Mean ±SD		34±25.94	40.82±27.24	29.26±22.79	28.48±22.52	108	12.00	0.08
**Pituitary adenomas complications**								
Cavernous invasion No (%) (112)		46 (41.1)	15/39 (38.46)	17/42 (40.47)	12/27 (44.44)	2/3 (66.66)	0	0.40
Chiasma compression No (%) (109)		28 (25.7)	7/39 (17.94)	11/39 (28.20)	10/27 (37.03)	0	0	0.21
Hypopituitarism No (%) total (103)		25 (24.3)	6/38 (15.78)	9/38 (23.68)	10/24 (41.66)	0	0	0.17
Hypogonadism No (%) total (70)		49 (70)	14/23 (60.68)	27/31 (87.09)	8/15 (53.3)	0		0.51
Diabetes insipidus No (%) total (105)		5 (4.8)	0	1/41 (2.43)	4/23 (17.39)	0	0	0.31
Apoplexy No (%) total (113)		3 (2.65)	0	1/43 (2.32)	2/27 (7.40)	0	0	0.47
CSF^€^ rhinorrhea No (%) total (113)		2 (1.76)	0	2/43 (4.65)	0	0	0	0.50
**Mode of therapy**								
No therapy No (%)		10/113 (8.84)	0	2/43 (4.65)	7/27 (25.92)	0	1 (100)	0.0001
Medical therapy No (%)		52/113 (46.01)	22/39 (56.41)	25/43 (58.13)	4/27 (14.81)	1/3 (33.33)	0	0.003
Surgical therapy total No (%)		45/113 (39.82)	17/39 (43.58)	12/43 (27.90)	14/27 (51.85)	2/3 (66.66)	0	0.201
Surgical approach	TSS ^¥^ No (%)	32/45 (71.11)	12/17 (70.58)	10/12 (83.33)	8/14 (57.14)	2/2 (100.0)	0	0.61
	TCS ^§^ No (%)	4/45 (8.88)	0	2/12 (16.66)	2/14 (14.28)	0	0	
	TSS and TCS No (%)	3/45 (6.66)	2/17 (11.76)	0	1/14 (7.11)	0	0	
	Two TSS No (%)	5/45 (11.11)	3/17 (17.64)	0	2/14 (14.3)	0	0	
	Two TCS No (%)	1/45 (2.22)	0	0	1/14 (7.14)	0	0	
Radiosurgery No (%)		14/113 (12.38)	4/39 (10.25)	8/43 (18.60)	2/27 (7.40)	0	0	0.60
Combination of modalities of therapy (medical, surgical, radiosurgery) No (%)		8/113 (7.07)	4/39 (10.25)	4/43 (9.30)	0	0	0	0.96

ș SD = standard deviation, έ BMI = body mass index, £ DM = diabetes mellitus, ¥ TSS= transsphenoidal hypophysectomy, § TCS = transcranial hypophysectomy, €CSF= cerebrospinal fluid

NB: Some patients have more than one modality of treatment.

### Clinical characteristics and treatment modalities

Obesity was more frequently observed in patients with prolactinoma than other adenomas. Type 2 diabetes mellitus (T2DM) was observed in 22.2% of the enrolled patients. The complications seen were cavernous invasion in 41.1% of cases, chiasma compression in 25.7%, hypopituitarism in 24.3%, hypogonadism in 70.0%, diabetes insipidus in 4.8%, pituitary apoplexy in 2.65%, cerebrospinal fluid rhinorrhea in 1.76%, and ventricular shunt for hydrocephalus in 0.88% of cases. None of these had a statistically significant association with any of the types of adenomas. Co-secretory functioning adenomas were seen in 6.19% of cases, secreting both GH and PRL, but no immunohistochemistry confirmation was done.

Among the enrolled patients, 8.84% did not receive any therapy, with a higher proportion observed in NFPA patients (25.92%) and lower proportions in prolactinoma patients (4.65%) and TSH-omas patients (1 patient).

Medical treatment was used in 52/113 (46.0%) patients of all adenoma cases. It was the primary modality of treating both prolactinomas in 58.13% (dopamine agonist) and acromegaly in 56.41% (long-acting somatostatin receptor ligand) of cases. Surgical treatment was performed in 45/113 (39.82%) patients, stereotactic radiosurgery in 14/113 (12.38%) patients, transsphenoidal hypophysectomy in 32/45 (71.11%) patients, transcranial hypophysectomy in 4/45 (8.88%) patients, and combination of both approaches in 3/45 (6.66%) patients for pituitary adenomas. 5/45 (11.11%) patients treated surgically required a second transsphenoidal, and 1/45 (2.22%) patients required 2 transcranial surgeries done. A combination of medical, surgical, and/or radiosurgery was seen in 8/113 (7.07) patients.

### Adenoma volume measurements and correlations

Table 2 shows a statistically significant difference in mean pre-treatment volume across all pituitary adenoma types except for Cushing disease and TSH-omas, with the ellipsoid method yielding larger volumes than the perimeter method (p-value <0.05). However, no significant differences were observed in mean post-treatment volumes, except for prolactinomas. Table 3 shows strong positive and significant correlations between the ellipsoid and the perimeter methods in the pre-treatment and the post-treatment volume across all pituitary adenomas.

**Table 2:** Mean measurements of the initial volume and the second tumor volume using ellipsoid and perimeter measurements for each adenoma type

Adenoma type	Acromegaly Mean± SD (cm^3^)	Prolactinoma Mean±SD (cm^3^)	NFPA Mean± SD (cm^3^)	Cushing disease Mean± SD (cm^3^)	TSH-oma Mean±SD (cm^3^)
Volume 1 by ellipsoid	8.85±16.60	7.74±7.09	13.33±18.08	1.43±0.97	4.24
Volume 1 by perimeter	4.93±5.68	6.17±5.92	9.61±13.38	1.11±0.67	3.456
p value	0.05	0.0001	0.01	0.38	
Volume 2 by ellipsoid	7.17±17.22	6.60±10.04	7.21±13.05	0.89	2.00
Volume 2 by perimeter	4.96±9.28	4.73±7.12	6.64±10.15		1.554
p value	0.24	0.011	0.69		

**Table 3:** Mean differences and correlations between the ellipsoid and perimeter measurements of adenoma volumes before (Vol 1) and after treatment (Vol 2) among different types of adenomas using paired t-test

Treatment modality		No	Mean difference ±SD	p value*	95^th^ CI interval	R coefficient	p value**
**Acromegaly**							
Pre-treatment ^§^	Vol 1	37	4.00±12.15	0.05	-0.05-8.05	0.86	<0.0001
Medical treatment	Vol 2	14	0.17±0.96	0.51	-0.38-0.72	0.90	<0.0001
Surgery alone	Vol 2	4	0.58±0.98	0.31	-0.98-2.15	0.988	0.01
Combination of therapy including radiosurgery	Vol 2	8	5.95±15.37	0.31	-6.90-18.81	0.99	<0.0001
**Prolactinoma**							
Pre-treatment	Vol 1	43	1.56±2.31	<0.0001	0.85-2.27	0.95	<0.0001
Medical treatment	Vol 2 ^£^	18	1.44±3.00	0.05	-0.04-2.93	0.85	<0.0001
Combination of therapy including Surgery and radiosurgery	Vol 2	11	2.19±4.88	0.166	-1.07-5.47	0.97	<0.0001
**NFPA**							
Pre-treatment	Vol 1	27	3.71±7.62	0.01	0.69-6.72	0.92	<0.0001
Medical treatment	Vol 2	4	0.52±0.79	0.27	-0.73-1.78	0.97	0.02
Surgery including radiosurgery	Vol 2	12	0.58±6.54	0.76	-3.57-4.73	0.90	<0.0001

* p value for the mean ± SD difference between ellipsoid and perimeter by paired t-test.

** p value for the correlation between ellipsoid and perimeter.

§ In acromegaly, one patient was missing the first volume by ellipsoid, and one patient was missing the perimeter volume.

£ in prolactinoma and NFPA patients, the post-treatment volume did not match the pre-treatment volume due to missing the second volume.

A paired Student's t-test revealed no significant difference in the mean post-treatment pituitary adenoma volume measurements when using the ellipsoid and perimeter methods across different treatment modalities. However, significant differences were observed in the pre-treatment volume measurements between the two methods for NFPA and prolactinoma.

### Biochemical control and adenoma size reduction

The percentage of patients with acromegaly who achieved adenoma size reduction using medical therapy alone was 66.66%, while the percentage of patients who achieved biochemical control (serum GH≤2.5 ng/mL) was 62.50% (Table 4). A combined reduction in adenoma size and biochemical control was achieved in 53.33% of cases. For patients with prolactinoma, dopamine agonists resulted in tumor size reduction in 88.88% of cases, and 63.63% achieved biochemical control. The combination of adenoma size reduction and biochemical control was achieved in 58.82% of patients.

**Table 4:** Tumor shrinkage and hormonal control on medical therapy among functioning adenomas patients

Adenoma type	No (%) of with tumor shrinkage (≥ 1mm)	No (%) with biochemical control	Both biochemical and tumor shrinkage
Acromegaly	10/15(66.66)	10/16(62.50)	8/15(53.33)*
Prolactinoma	16/18(88.88)	14/22(63.63)	10/17(58.82)**

* 2.5 ng/mL represents the lower limit cutoff for the degree of biochemical remission for acromegaly.

** 30 ng/mL represents the lower limit cutoff for the degree of biochemical remission for prolactinoma.

There was no correlation between the percentage of volume variability (decrease or increase in size) and hormonal control by utilizing both ellipsoid and perimeter methods in functioning adenomas (Table 5).

**Table 5:** Correlation between volumetric change in percentage (increase or decrease) between the ellipsoid and the perimeter method in relation to biochemical hormonal control in functioning pituitary adenomas using paired t-test

Adenoma type	Measurement method	Unit measurement limits	Volume change (% ± SE)	p value
Acromegaly	Ellipsoid	<2.5 * ng/mL	18.0±7.32	0.32
	Perimeter	<2.5 ng/mL	4.9±9.25	0.33
	Ellipsoid	≥2.5 ng/mL	5.7±9.89	0.17
	Perimeter	≥2.5 ng/mL	-22.7±20.0	0.23
Prolactinoma	Ellipsoid	<20** ng/mL	22.7±18.8	0.70
	Perimeter	<20 ng/mL	22.4±19.9	0.72
	Ellipsoid	≥20 ng/mL	30.3±10.0	0.86
	Perimeter	≥20 ng/mL	26.5±13.21	0.86

* 2.5 represents the lower limit cut-off for the degree of biochemical improvement in acromegaly

** 20 ng/mL r represent the lower limit cut-off for the degree of biochemical response for prolactinomas

## DISCUSSION

This study conducted at the Faiha Specialized Diabetes, Endocrine, and Metabolism Center (FDEMC) in Iraq is the second study on pituitary macroadenomas conducted in the country and the first one to specifically focus on the measurement of pituitary adenoma volume [12]. Measuring pituitary adenoma volume has gained popularity in the last decade, but it should not lead endocrinologists to modify their decision on the optimal approach to therapy. Although the maximal dimension or the largest tumor diameter has been adopted for many years to measure pituitary volume, it is associated with enormous errors [8].

Both methods (the ellipsoid and perimeter) are observer-dependent. The ellipsoid is best described and applied to more spherical-shaped adenomas, while the perimeter method is more useful in adenomas with irregular margins, especially for those after surgery where the remnant is irregular compared to the more circumscribed tumor before surgery. Therefore, the accuracy is largely dependent on the timing of the measurement [18]. Measuring volume via the slice-by-slice or perimeter method depends on the Cavalieri principle, which is applied in irregularly shaped masses, and here the perimeter method is the method of choice [19].

The mean age of patients in this study was 42.16±13.5 years, which is comparable to the mean age in a previous study done at the same center (FDEMC) of 42.5±14.9 years and also comparable to a retrospective Iranian study on pituitary adenomas, which was 40 years [12, 20]. A total of 113 patients were included in this study, which is slightly larger than the sample size of 99 patients in a study conducted by Davies BM *et al*. [18] and higher than the sample size of 94 patients in a study by Chi-Cheng Chuang *et al*. [14]. The gender distribution in our study showed an equal representation, with men comprising half of the enrolled patients and a male-to-female ratio of 1:1 [12, 20].

Several studies have documented and observed an increased frequency of obesity among prolactinoma patients on their initial presentation. In a study by Santos Silva *et al*. (2001) on 35 patients with prolactinoma before and after treatment with a dopamine agonist, they observed increased obesity prevalence among their patients on initial presentation with significant improvement in insulin resistance after treatment [21]. We found a significant prevalence of obesity among prolactinoma patients during their initial presentation, compared to other groups of pituitary adenomas. Obesity in prolactinoma patients at diagnosis raises important considerations regarding the potential interplay between prolactinoma and metabolic disturbances.

Cavernous sinus invasion was reported in less than half of the patients in the present study, while it was 38.14% in the study by Jianxing Niu *et al*. [7]. The invasion was rated qualitatively preoperatively according to Hardy and Knosp classification but not subjected to detailed classes, and no informative data was obtained from the intraoperative or postoperative pathological examination. A similar percentage was found in studies done in Austria [7, 22].

The reported chiasmal compression in this study was seen in a quarter of patients on radiological imaging with or without visual complaint. A systematic review revealed a wide range (14-84%) of chiasmal compression in patients with macroadenoma. That chiasmal compression was seen more in NFPA due to delays in the diagnosis [23].

Our study also underreported the chiasmal compression because the routine ophthalmological assessment was not feasible for all patients. In an Iranian study, chiasmal compression was seen in 74.4% of patients, while in an Italian study, chiasmal compression was seen in 27% of patients [20, 24].

This study showed the percentage of hypopituitarism in one-fifth of patients and hypogonadism in two-thirds of patients. In comparison, Nomikos P. *et al*. from Germany reported that the percentage of post-operative hypopituitarism was 85% in TSS and 86% in TCS. Half of those patients remained unchanged, while one-third improved and one-fifth normalized, and hypogonadism was found in 19% of patients [25].

Diabetes insipidus was transient in less than one-quarter of patients as a surgical complication and only became permanent in 0.9-2%. It was more frequently observed in microadenomas, transiently occurred in Cushing disease, and was unrelated to a second surgery in a Virginia study [26]. We reported a higher incidence of diabetes insipidus in this study (4.4%). Pituitary apoplexy was rare in our study, similar to previous literature [27].

In this study, the mean initial adenoma volume measured by the perimeter and ellipsoid methods for acromegaly was nearly equal to the mean initial volume measured by the same methods as Davies *et al*. in 2016 on pituitary tumor size [14].

For NFPA, the mean initial adenoma volume in this study was comparable to a volumetric comparison study done in 2010 at Chang Gung Memorial Hospital in Taiwan, where the mean adenoma volume was 12.09 [28].

In terms of volume measurement, the ellipsoid method tended to yield larger volume measurements compared to the perimeter method. This finding aligns with previous studies highlighting the superiority of the perimeter method in assessing volume, although it is more time-consuming [14, 15].

**Figure 2:**
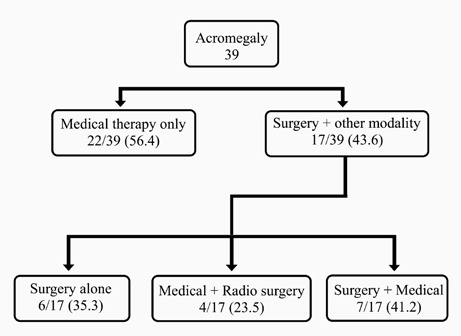
Management of somatotroph adenomas (Acromegaly)

**Figure 3:**
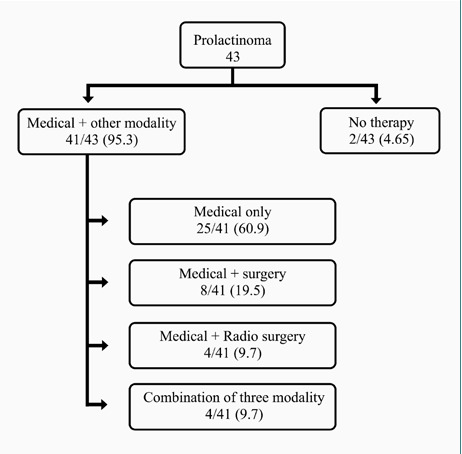
Management of lactotroph adenomas

**Figure 4:**
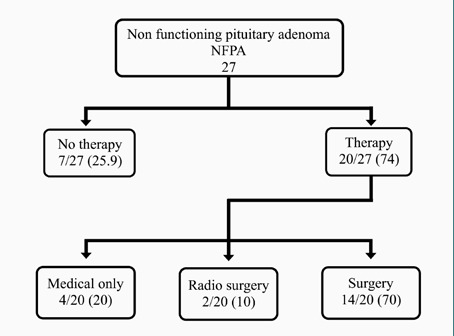
Management of NFPA

In contrast to the previous guideline on acromegaly, which recommended surgery as the initial treatment approach, our study employed somatostatin receptor ligands as the primary mode of therapy for the first 3-6 months, depending on the individual patient and adenoma characteristics (Figure 2). This approach was chosen due to the ongoing learning curve of our pituitary surgeons, as they have recently started to improve their surgical techniques [29]. Medical treatment was the primary mode of therapy for functioning adenomas, with more than half of patients with acromegaly and prolactinomas receiving some form of medical therapy, while surgery constituted seventy percent of NFPA management (Figures 3, 4).

The results of the second surgery, radiosurgery, and medical therapy, were comparable to those reported in a previous study conducted in Switzerland [30].

The two methods exhibited a positive, strong, and significant correlation between volumes measured by the ellipsoid and perimeter methods for the first and second volumes. The Pearson correlation coefficient (r) was 0.95 and 0.93 (p<0.0001) for the overall types of adenomas and each adenoma. This finding is consistent with the findings of Qasim Al Hinai *et al*. in Canada 2010. In their series, which included 38 patients, a comparison between manual and semi-automated volumetric measurement of pituitary adenomas showed a strong and significant Pearson correlation coefficient (r) of 0.969 (p<0.0001) [9]. Furthermore, our results are consistent with Davies *et al*., who reported a strong correlation between the ellipsoid and perimeter method (R^2^=0.99, p<0.0001) in their series of 99 consecutive patients with pituitary adenomas. The overall mean difference was very small and comparable to the mean difference of this study in post-treatment volume for each adenoma type [14].

A recent study by Chuang *et al*. from Taiwan investigated different volumetric methods for measuring pituitary adenoma volume in 94 patients. They compared the ellipsoid method using OsiriX software and found no significant difference in the first volume compared to the standard 3D slicer software. However, a significant difference was observed in the second volume (post-treatment volume), indicating that the slice-by-slice method was more precise for post-treatment volume measurements due to the irregularity of the tumors [18].

We also observed a significant difference between the two methods in the pre-treatment volume among different types of adenomas. However, no difference was found in the post-treatment volume when considering different therapeutic modalities.

Regarding acromegaly, more than half of the patients who received medical therapy alone achieved tumor volume reduction, and 62.50% achieved biochemical or hormonal control. These findings align with a meta-analysis conducted in 2012, which reported a 53% tumor shrinkage rate using somatostatin receptor ligands [31].

For patients with prolactinoma, we found that 88.88% of those on medical therapy alone experienced a reduction in tumor volume. Additionally, 63.63% of patients achieved biochemical control (using 30 ng/mL as the lower limit cutoff for biochemical remission), and 58.82% achieved both volume reduction and biochemical remission simultaneously. Data from a study by Colao *et al*. in 2000 on the effect of cabergoline treatment on prolactinoma tumor reduction showed that the percentage of patients who achieved normal prolactin with no previous treatment was 80%, and 53% in resistant patients. Volume reduction at the 3-year follow-up ranged from 50-80% according to the responsiveness to the cabergoline [32]. The combined adenoma size reduction with biochemical improvement seems to be better with prolactinoma than in acromegaly in this study because of the longer duration of follow-up, as seen in Table 1.

In our study, there was no correlation between reduced or increased tumor volumes and the degree of hormonal control for the given functioning adenomas. Therefore, tumor reduction or shrinkage should not necessarily be followed by hormonal control. One potential explanation is that adenomas may enlarge after discontinuing somatostatin receptor ligand therapy. Additionally, certain cases may result in a partially empty sella, with remaining active residual tumors. Moreover, resistant or refractory adenomas may persistently grow despite undergoing medical therapy [33]. The literature shows that the correlation between prolactinoma size and serum prolactin is not always linear, particularly in smaller prolactinoma tumors where this relationship may be less clear [34].

Regarding the limitations of our study, there were instances where second volume measurements to assess treatment response were missed. Additionally, the duration of follow-up was relatively short to fully evaluate the response to medical therapy. Furthermore, there is currently no software-automated method available that can be considered the gold standard for assessing pituitary adenoma volume.

## CONCLUSION

Both the ellipsoid and perimetric methods showed strong and positive correlations for pituitary adenoma volume measurements. However, the ellipsoid method tended to overestimate tumor volume. We found no correlation between adenoma size reduction and the degree of biochemical response in functioning adenomas.

## Personal thanks

We would like to express our sincere appreciation to the Radiology unit at Faiha General Hospital for their invaluable support and assistance throughout this study. Their dedication and expertise in conducting the necessary imaging scans were instrumental in the successful completion of the research. Furthermore, we extend my gratitude to the medical staff at Faiha Specialized Diabetes, Endocrine, and Metabolism Center (FDEMC), particularly the laboratory personnel, for their continuous support and collaboration.
